# A Beautiful Handle

**Published:** 1898-06

**Authors:** 


### A Beautiful Handle.

We have just received from Dr. E. C. Moore & Sons, of Detroit, a beautiful carrier for nerve broaches, dressing needles, etc. It is so constructed as to hold anything firmly put within its grasp. It is an instrument which every skillful operator would appreciate.

